# Proteomic Profiling of Iron Overload-Induced Human Hepatic Cells Reveals Activation of TLR2-Mediated Inflammatory Response

**DOI:** 10.3390/molecules21030322

**Published:** 2016-03-17

**Authors:** Xiang Li, Shiwei Li, Mize Lu, Guohua Yang, Yunfeng Shen, Xin Zhou

**Affiliations:** 1 Wuxi Medical School, Jiangnan University, Wuxi 214122, China; 2 Key Laboratory of Carbohydrate Chemistry & Biotechnology Ministry of Education, Jiangnan University, Wuxi 214122, China; shiweili099@sohu.com; 3 School of Biotechnology, Jiangnan University, Wuxi 214122, China; 4 Department of Hematology, Wuxi People’s Hospital, Wuxi 214023, China; lmzmedsci@163.com (M.L.); wxygh@medmail.com.cn (G.Y.); yfshen21cn2012@hotmail.com (Y.S.)

**Keywords:** iron overload, proteomics, TLR2, IL-6, NF-κB

## Abstract

Background: Hepatic iron overload is common in patients who have undergone hematopoietic cell transplantation (HCT) and may predispose to peri- and post-HCT toxicity. To better reveal more molecules that might be involved in iron overload-induced liver injury, we utilized proteomics to investigate differentially expressed proteins in iron overload-induced hepatocytes *vs.* untreated hepatocytes. Methods and Results: HH4 hepatocytes were exposed to ferric ammonium citrate (FAC) to establish an *in vitro* iron overload model. Differentially expressed proteins initiated by the iron overload were studied by two-dimensional liquid chromatography tandem mass spectrometry (2D-LC-MS) analysis. We identified 93 proteins whose quantity statistically significantly changes under excess hepatocyte iron conditions. Gene Ontology (GO) analysis showed that these differentially expressed proteins in HH4 cells are involved in various biological process including endocytosis, response to wounding, di-, trivalent inorganic cation homeostasis, inflammatory response, positive regulation of cytokine production, and *etc.* Meanwhile, proteomics data revealed protein level of TLR2 and IL6ST significantly increased 7 times and 2.9 times, respectively, in iron overloaded HH4 cells. Our subsequent experiments detected that FAC-treated HH4 cells can activate IL6 expression through TLR2-mediated inflammatory responses via the NF-κB pathway. Conclusions: In this study, we demonstrated that iron overload induced hepatocytes triggering TLR2-mediated inflammatory response via NF-κB signaling pathway in HH4 cells.

## 1. Introduction

Allogeneic hematopoietic cell transplantation (HCT) has been developed to cure clonal disorder diseases, such as myelodysplastic syndromes (MDS) [1,2]. Preclinical and limited clinical data suggest that iron overload related to anemia, red blood cell transfusions and, possibly other factors, may be associated with hepatic injury in patients undergoing HCT [1,3]. Iron overload will lead to tissue damage and even iron poisoning because cells lack an effective way to dispose of the excessive iron. The liver is the major organ for iron storage and patients with chronic iron overload in the liver are at significantly higher risk of further developing liver fibrosis, cirrhosis, and hepatocellular carcinoma [4].

Our previous work have shown that ferric ammonium citrate (FAC)-induced iron overload in HH4 cells was capable of increasing apoptosis through an iron-generated ROS-activated signaling pathway [5], however the cellular consequences and process of iron overload in hepatocytes are still not very clear and need to be further elucidated. Therefore, the current study was aimed at assessing protein expression changes initiated by excess iron using a proteomic approach. We attempted to characterize the new “molecules” that are involved in inflammatory response induced by iron overload in HH4 cells. Our proteomics data revealed protein levels of Toll-like receptor 2 (TLR2) and IL6ST significantly increased seven times and 2.9 times, respectively, in iron overload HH4 cells. The subsequent results detected that FAC-treated HH4 cells can activate IL6 expression through TLR2-mediated inflammatory responses via NF-κB signaling pathway in HH4 cells.

## 2. Results

### 2.1. Overview of Proteomic Analysis (2D-LC-MS)

**Figure 1:**
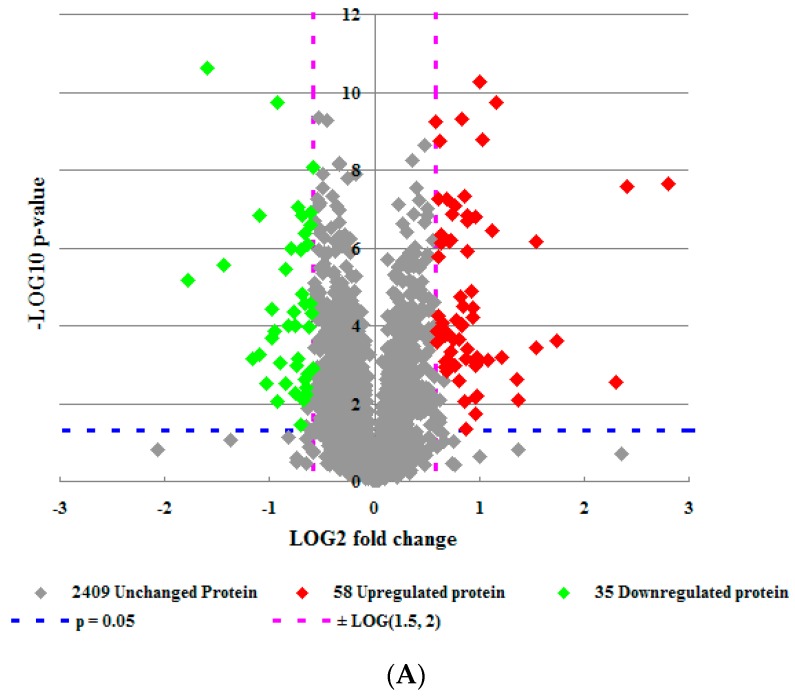

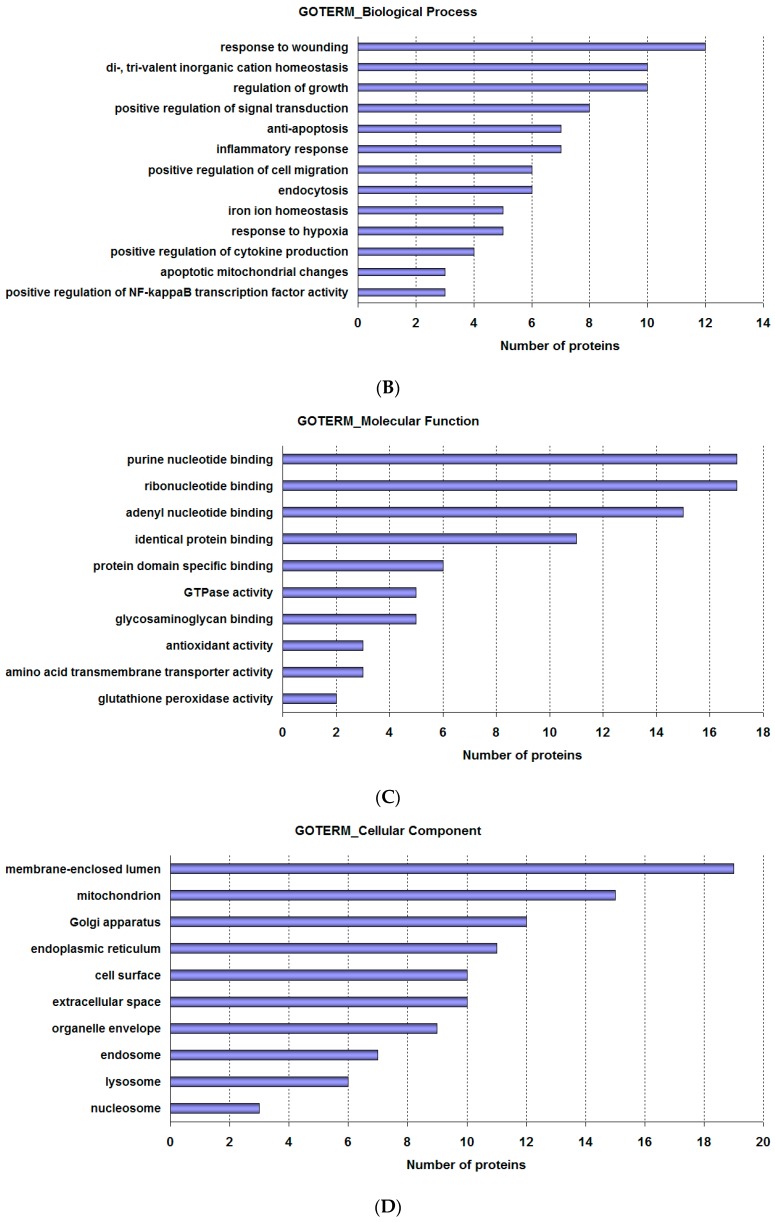

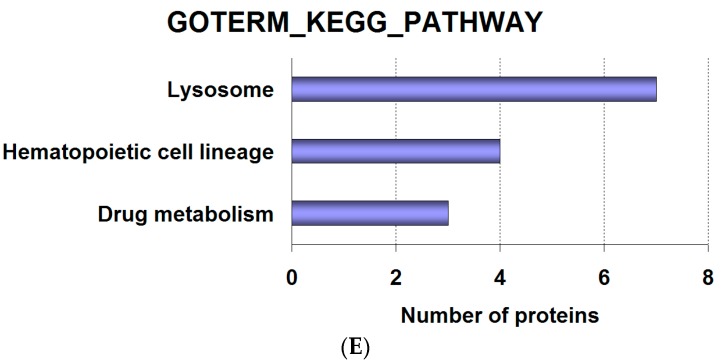
Differential proteomic analysis in HH4 cells. (**A**) Volcano plot of 2502 differentially expressed proteins between iron overload treatment group and control group in HH4 cells. (**B**–**D**) Functional classification of differentially expressed proteins using SWISS-PROT database based on universal GO annotation terms. Proteins shown were linked to at least one annotation term within the GO biological process (**B**); molecular function (**C**); and cellular component (**D**) categories; (**E**) KEGG pathway analysis.

To gain insight into the effect of FAC-induced iron overload on HH4 cells, 2D-LC-MS were carried out to compare the protein profiles of HH4 cells stimulated by FAC at concentrations of 0 and 5 mM, respectively. A total of 2502 unique proteins were identified in both of the two independent replicate experiments. Among these, expression of 58 proteins was higher (more than 1.5-fold) and expression of 35 proteins was lower (less than 0.67 fold) in FAC-treatment HH4 cells compared to untreated cells (Figure 1A and Table 1). Next, we analyzed the main properties of these identified differentially expressed proteins using the Database for Annotation, Visualization and Integrated Discovery (DAVID) gene bioinformatic resources. The results showed these differentially expressed proteins are involved in various biological process mainly including response to wounding, di-, tri-valent inorganic cation homeostasis, regulation of growth, positive regulation of signal transduction, anti-apoptosis, inflammatory response, *etc.* (Figure 1B and Table 2).

In addition, Gene Ontology (GO) analysis revealed that the most common molecular functions were purine nucleotide binding, ribonucleotide binding, adenyl nucleotide binding and identical protein binding (Figure 1C). On the other hand, the major cellular component categories were membrane-enclosed lumen, mitochondrion, golgi apparatus, endoplasmic reticulum and cell surface (Figure 1D). Subsequently, on the basis of KEGG pathways, we found that highly significant enrichment was seen in several pathways mainly including lysosome, hematopoietic cell lineage and drug metabolism (Figure 1E).

### 2.2. Analysis of Proteomics Data

Next, we analyzed 93 differentially expressed proteins using Ingenuity Pathways Analysis (IPA) (Ingenuity Systems, Redwood, CA, USA, www.ingenuity.com). The metabolic and canonical pathways as well as the interconnecting proteins were generated.

**Figure 2:**
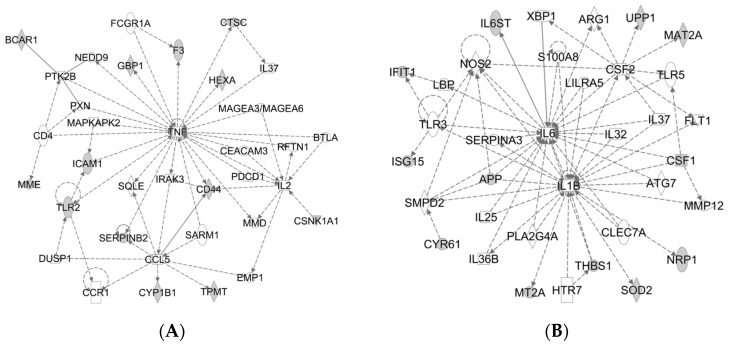
Biological pathways analysis of differentially expressed proteins in HH4 cells. (**A**) Top network functions of cell-to-cell signaling and interaction, inflammatory response, cellular movement and (**B**) immune cell trafficking using Ingenuity Pathways Analysis (IPA). Solid lines: direct known interactions. Dashed lines: suspected or indirect interactions. White: proteins known to be in the network but not identified in our study.

The results showed that 13 proteins were involved in cell-to-cell signaling and interaction, inflammatory response and cellular movement pathway (Figure 2A). Twelve proteins were involved in cellular movement, inflammatory response and immune cell trafficking pathway (Figure 2B). These findings indicate that HH4 cell proteomes were markedly shifting after FAC overload treatment.

### 2.3. Validation of LC-MS/MS Results by RT-PCR

**Figure 3:**
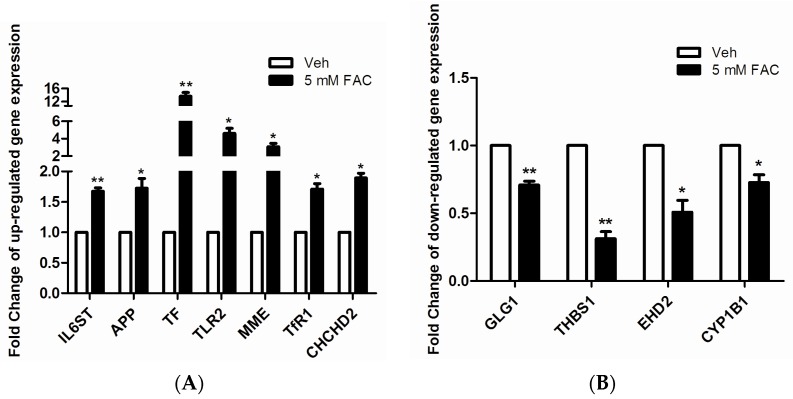
Confirmation of mass spectrometry-determined protein using RT-PCR technique. (**A**) Gene expression analysis of seven kinds of up-regulated proteins randomly selected from 93 kinds of differentially expressed proteins; (**B**) Gene expression analysis of four kinds of down-regulated proteins randomly selected from 93 kinds of differentially expressed proteins. The expression of genes was classified as up-regulated if expression levels were >1.5-fold and as down-regulated if levels were <0.67-fold, relative to control HH4 cells. Expression of β-actin was used as internal control. Results are shown as mean ± SEM of three independent experiments. Statistically significant differences are labeled as * *p* < 0.05 and ** *p* < 0.01, compared with the control groups, using unpaired Student’s *t* test.

In order to verify the accuracy of protein identifications made by LC-MS/MS, we randomly selected 11 proteins from 93 differentially expressed proteins to validate their mRNA levels. The results, as shown in Figure 3A, indicate when HH4 cells were treated with 5 mM FAC for 24 h, mRNA levels of gene IL6ST, APP, TF, TLR2, MME, TfR1 and CHCHD2 increased 1.7, 1.7, 13.6, 4.6, 3.1, 1.7, and 1.9 times, respectively compared to untreated HH4 cells. In addition, as shown in Figure 3B, the mRNA levels of GLG1, THBS1, EHD2 and CYP1B1 reduced 0.7, 0.3, 0.5 and 0.7 times, respectively in FAC-treated cells compared to untreated HH4 cells. Our findings are consistent with the results from proteomic mass spectroscopy.

### 2.4. FAC-Induced Iron Overload Affected Iron Homeostasis Related Proteins

**Figure 4:**
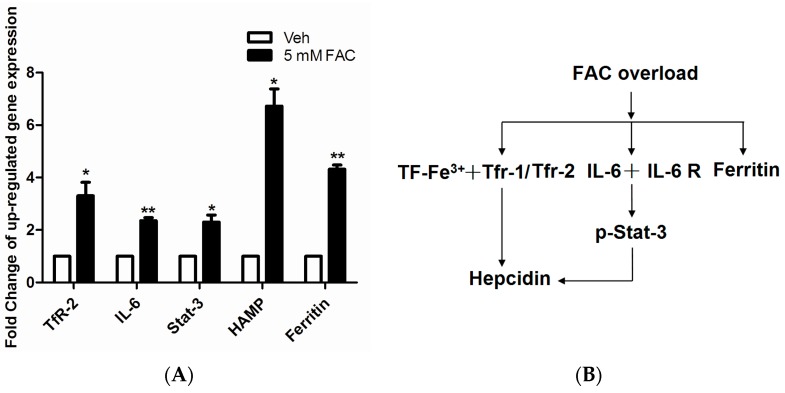
The effect of FAC overload on expression levels of iron homeostasis related gene. (**A**) FAC overload induced upregulation of iron homeostasis related protein expression. Expression of β-actin was used as internal control. Results are shown as mean ± SEM of three independent experiments. Statistically significant differences are labeled as * *p* < 0.05 and ** *p* < 0.01, compared with the control groups, using unpaired Student’s *t* test; (**B**) Schematic representation of the activation of Hepcidin regulation-associated signal transduction pathway in HH4 cells with FAC treatment.

After annotated the function of 93 differentially expressed proteins, we found the level of intracellular iron homeostasis related proteins increased significantly in FAC treated HH4 cells, such as IL6ST, TF and TfR1. It is well known that hepcidin is the center regulator to ensure iron metabolism in steady state. Therefore, we were interested in whether FAC-induced iron overload can affect expression of hepcidin and iron regulatory related proteins. As shown in Figure 4A, mRNA levels of gene TfR2, IL-6, STAT3, hepcidin and ferritin significantly increased after 24 h of exposure to 5 mM FAC in HH4 cells, suggesting FAC exposure facilitated TF-TfR2 and IL 6-STAT3 signaling pathway to further activate hepcidin expression (Figure 4B).

### 2.5. FAC Overload Triggered TLR2-Mediated Inflammatory Response

**Figure 5:**
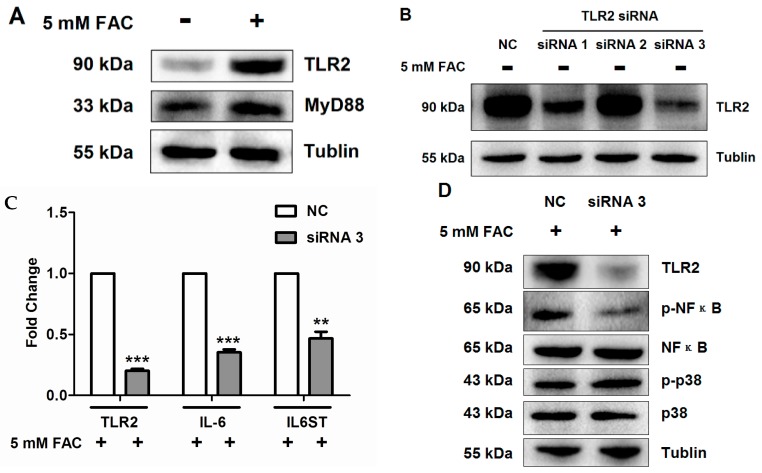
The activation of TLR2 pathway induced by iron overload in HH4 cells. (**A**) Protein expression of TLR2 and MyD88 were analyzed by western blot in HH4 cells after 24 h of 5 mM FAC treatment; (**B**) Inhibitory effects of siRNA for TLR2 in HH4 cells. Cells (10^5^ cells/well) were pre-treated with 60 nM siRNA for TLR2 for 48 h and then interference efficiency was estimated by detection of protein expression levels of TLR2 using western blot assay. NC = negative control siRNA; siRNA 1 = TLR2 siRNA (950 bp); siRNA 2 = TLR2 siRNA (1377 bp); siRNA 3 = TLR2 siRNA (1648 bp); (**C**) Effects of siRNA for TLR2 on transcriptional levels of IL-6 and IL6ST in HH4 cells. Cells (10^5^ cells/well) were pre-treated with 60 nM siRNA for TLR2 for 48 h. Then HH4 cells were treated with 5 mM FAC for 24 h and transcriptional levels of gene IL-6 and IL6ST via RT-PCR assay. Expression of β-actin was used as internal control. Results are shown as mean ± SEM of three independent experiments. Statistically significant differences are labeled as ** *p* < 0.01 and *** *p* < 0.001, compared with the control groups, using unpaired Student’s *t* test; (**D**) Effects of siRNA for TLR2 on NF-κB and p38 pathway in HH4 cells. Cells (10^5^ cells/well) were pre-treated with 60 nM siRNA for TLR2 for 48 h. Then HH4 cells were treated with 5 mM FAC for 24 h and the protein expression levels of p-NF-κB, NF-κB, p-p38 and p38 using Western blot assay. +: in the presence of 5mM FAC, - : in the absence of 5mM FAC.

In the foregoing analysis via GO and IPA, we noticed that TLR2 was involved in FAC-induced iron overload in HH4 cells (Figure 1B, Table 2, Figure 2A) and the mRNA level of TLR2 was also consistant with proteomics data (Figure 3A). We next investigated the protein level of TLR2 and its related pathway in FAC treated HH4 cells. As shown in Figure 5A, protein levels of TLR2 significantly increased in HH4 cells after 24 h of exposure to 5 mM FAC and its downstream factor, MyD88, was also significantly increased, indicating that FAC treatment activated the TLR2-MyD88 signaling pathway. In order to clarify the specific role of TLR2 in FAC treated hepatocytes, we next inhibited the endogenous TLR2 expression in HH4 cells using RNA interference.

As shown in Figure 5B, inhibition using siRNA significantly reduced protein levels of TLR2. In addition, inhibition of TLR2 markedly decreased the expression of IL-6 and IL6ST at transcriptional level in FAC-treated HH4 cells, compared to untreated cells (Figure 5C). Our data also indicated p-NF-κB but not p-p38 involving in activation of TLR2-IL6 pathway (Figure 5C,D).Taken together, these results suggested that TLR2-mediated inflammatory response triggered by FAC induced the expression of IL-6 through activating the NF-κB pathway rather than p38 pathway in HH4 cells.

## 3. Discussion

HCT is by far the most effective way to treat the clonal disorder disease [6]. However, patients commonly have an increase of nontransferrin-bound iron (NTBI) and show hepatic iron overload already before HCT or after transplant conditioning [7,8]. Excessive iron can stimulate the formation of ROS via the Fenton reaction [9], and progressive accumulation of ROS may damage mitochondrial and nuclear DNA through lipid peroxidation [10]. In our previous work, we have shown that iron overload can induce apoptosis via both extrinsic and intrinsic pathways in hepatic cells [5]. To our knowledge, the exact cellular consequences and the cellular processes of iron overload in hepatocytes are still not very clear. In this current study, we utilized proteomics technique to dissect more signaling pathways that related to hepatocytes iron overload.

The mass spectrum results showed FAC-induced HH4 cells contained 93 differentially expressed proteins and activated multiple pathways (Figure 1A and Table 1). Levels of protein ATP6V1H, which facilitates acidification of intracellular endosomes formed by transferrin/transferrin receptor-mediated endocytosis [11,12], increased 1.6 times (Table 1), while levels of protein EHD2, which plays an important role in regulating transferrin exit from ERC (endocytic recycling compartment) [13], decreased three times (Table 1) when HH4 cells were treated with 5 mM FAC at 24 h. These two significantly changed proteins indicate that HH4 cells may absorb a large number of extracellular iron ion via endocytosis.

Addition of excess iron inevitably led to iron dyshomeostasis and thus triggered differential expression of intracellular iron homeostasis associated proteins in HH4 cells (Figure 1B). Levels of protein TF (serotransferrin) which is mainly produced by hepatocytes and capable of binding excess iron ions and delivering it into cells increased sharply in FAC-overload HH4 cells (Table 1 and Figure 3A). Levels of protein IRP1, which is also known as cytoplasmic aconitate hydratase (ACO1), and is the trans-acting factor located in the cytoplasm that can bind with high affinity to RNA motifs related to iron homeostasis [14], decreased 0.5 times (Table 1) and levels of the protein ferritin, each molecule of which is capable of storing up to 4500 iron atoms [15], increased 4.4 times (Figure 4A) in FAC- treated HH4 cells. Meanwhile, our MS data displayed the expression of γ-secretase C-terminal fragment 59 and amyloid-like protein 2 were increased 3.3-fold and 2.9-fold in FAC treated cells, respectively (Table 1), suggesting that full length APP protein, which can stabilize surface FPN , may participate in the efflux of iron from HH4 cells [16]. Our MS data also showed the expression of metallothionein-2 increased 1.8 times but SOD2 expression decreased 0.6 times in iron-loaded HH4 cells. Metallothioneins (MTs), which are conserved in the animal kingdom, can chelate heavy and trace metals such as zinc, copper or iron through sulfur-based clusters, and therefore the main function of MTs is the regulation of homeostasis, like the protection against oxidative stress or metals [17]. While SOD2 (superoxide dismutase [Mn], mitochondrial) functioned as the antioxidant is known to scavenge the ROS, specifically the hydroxyl radical [18]. In a word, these significantly changed proteins indicate that iron overload induces biological process of di-, tri-valent inorganic cation homeostasis in HH4 cells (Figure 1B and Table 2).

In addition, hepcidin is undoubtedly the most central regulator of iron hemostasis, and our results also confirmed that FAC-induced HH4 cells showed evidently enhanced transcription levels of gene TF, TfR1/2, IL-6, IL6ST, STAT3 and hepcidin (Figure 4), indicating that hepcidin was involved in the re-regulation of iron homeostasis in iron overload-treated HH4 cells.

Mass spectral analysis also indicated the participation of other biological process including response to wounding, cell apoptosis and inflammatory response in FAC-induced HH4 cells. For example, thrombospondin-1 (THBS1) which is a matricellular glycoprotein first discovered in activated platelets that plays an important role in the process of wound healing [19], decreased 0.47 times (Table 1 and Figure 3B), while the expression of endophilin-B1, which is involved in the regulation of apoptosis helping the maintenance of mitochondrial morphology and autophagy [20], was apparently increased in HH4 cells after iron overload treatment (Table 1). Among these, the most interestingly finding is the expression of inflammation mediator TLR2 increased markedly in FAC- treated HH4 cells (Table 1). Inflammation is a fundamental biological process that stands at the foreground of acute and chronic pathological conditions [21]. Toll-like receptors (TLRs) discovered in the 1990s are a family of very similar proteins containing leucine-rich repeats and their activation triggers a signaling cascade which leads to the production of cytokines/chemokines, further initiating an inflammatory response [22]. So far 13 TLRs have been identified, 10 human TLRs (TLR1-10), and 12 mouse TLRs (TLR1-9, TLR11 and TLR12) [23]. It is well known that the acute phase response to infection and inflammation can cause alterations in iron homeostasis, thereby reducing iron supplies to pathogens. A recent study found that the acute inflammatory condition mediated by TLR2 and TLR6 induced rapid hepcidin-independent hypoferremia through decreasing the messenger RNA and protein expression of ferroportin (FPN) in mice injected with TLR ligands [24]. Although TLR2 has been implicated in the response to infection mediated by TLR2 ligands, includes molecules with diacyl and triacylglycerol moieties, proteins and polysaccharides, scanty data is available on the direct relationship between TLR2 and iron overload. Therefore, our next research validated TLR2 mRNA and protein expression in FAC treated HH4 cells (Table 1, Figure 3A and Figure 5A), which is consistent with proteomics data. In addition, our experiment indicated that inhibition of TLR2 by siRNA inactivated IL-6 expression via NF-κB pathway in FAC treated hepatocytes (Figure 5).

## 4. Experimental Section

### 4.1. Cell Culture

The non-transformed human hepatocyte cell line HH4 was maintained as described previously [7]. Briefly, HH4 cells were maintained in Williams’ medium E (Gibco Laboratories, Grand Island, NY, USA), supplemented with 10% fetal bovine serum (FBS), 0.1% ITS, dexamethasone (0.04 μg/mL) (Sigma Chemical Co., St. Louis, MO, USA), gentamycin (50 μg/mL) (Sigma Chemical Co.), HEPES (20 mM) and sodium pyruvate (1 mM) (Gibco Laboratories).

### 4.2. In Vitro Iron Overload Model

The *in vitro* iron overload model was established as previously described [5]. Briefly, HH4 cells were seeded in 15 cm culture dishes at 1 × 10^5^ cells/mL, incubated overnight at 37 °C in 5% CO_2_ atmosphere, and then treated with or without 5 mM FAC (Sigma Chemical Co.) for 24 h. The experiments were repeated three times.

### 4.3. Proteomics Analysis

#### 4.3.1. Cell Lysis and Protein Digestion

HH4 cells were washed three times with PBS buffer, harvested by trypsinization, and centrifuged at 1000× *g* for 5 min. After the addition of T-PER Reagent (Thermo Scientific, Rockford, CA, USA), PMSF (1 mM) and 0.1% aprotinin, the cell pellets were lysed for 30 min on ice and centrifuged at 13, 800× *g* for 15 min. Then the supernatant was transferred into new tubes. The Broadford assay was used to determine the protein concentration (Beyotime Institute of Biotechnology, Shanghai, China). Proteins were reduced with 10 mM dithiothreitol (DTT) at 56 °C for 45 min and then alkylated with 10 mM iodoacetamide (IAM) (Sigma Chemical Co.) for 20 min at room temperature in the dark. The sample was diluted four times with 40 mM ammonium bicarbonate buffer. Sequencing grade trypsin (Promega, Madison, WI, USA) was added in a 1:50 (*w*/*w*) ratio and the incubation was carried out overnight at 37 °C. After an over-night digestion peptides were recovered from the filter. Peptides were desalted with Oasis 1cc cartridges and flow through from Oasis cartridge was desalted with Sep-Pak C18 1cc (Waters, Milford, MA, USA). Elutions from Oasis and Sep-Pak were combined together and concentrated in SpeedVac.

#### 4.3.2. Nanoflow LC-MS/MS

Peptide digests dissolved in loading buffer (0.1% trifluoroacetic acid—TFA) were loaded onto an in-house packed 20 cm capillary column with 3 µm Reprosil-Pur C18 beads (Dr. Maisch GmbH, Ammerbuch, Germany) using an EASY-nLC 1000 system (Thermo Scientific). Running buffer A was 0.1% TFA in water and running buffer B was 0.1% TFA in ACN (acetonitrile). The total gradient was 120 min and the flow rate started at 300 nL/min. The detailed gradient was 6% ACN with a linear increase to 30% ACN over 105 min followed by 4 min linear increase to 90% ACN. MS data were acquired using a data-dependent top-20 method on Q Exactive (Thermo Scientific, Bremen, Germany). Spray voltage was set to 2.0 kV, S-lens RF level at 60, and capillary temperature at 275 °C. Full scan resolutions were set to 60,000 at *m*/*z* 200 and AGC (automatic gain control) was 3 × 10^6^ with a maximum fill time of 20 ms. The range of full mass was set to 350–1500 *m*/*z*. MS2 scan resolutions were set to 15,000 at *m*/*z* 200 and AGC was 5 × 10^4^ with a maximum fill time of 45 ms. Isolation width was set at 1.6 Th. A fixed first mass of 110 was used. Normalized collision energy was set at 27. Peptide match was set to “preferred” and isotope exclusion was on. Precursor ions with single, un-assigned, charge states were removed from fragmentation selection [25,26,27].

#### 4.3.3. Data Analysis

All data were analyzed with the MaxQuant version 1.5.1.12 [28], with the Andromeda search engine [29]. The false discovery rate (FDR) was set at 1% for protein, peptide spectrum match. Peptides were required to have a minimum length of six amino acids and a maximum mass of 10,000 Da. Fragmentation spectra were searched by Andromeda in the Uniprot human database (version 201502; 90,300 entries) combined with 262 common contaminants [29]. Enzyme specificity was set as C-terminal to arginine and lysine and a maximum of two missed cleavages. Second peptides search was enabled. For proteome identification, carbamidomethylation (C) was set as a fixed modification. deamidation (NQ) and oxidation (M) were set as variable modifications. To identify significantly regulated proteins, LFQ intensities were used. Proteins with *p*-value < 0.05, fold change > 1.5 or < 0.67 were recognized as significant change.

### 4.4. Realtime-PCR

Total RNA was extracted using RNA pure Tissue Kit (Cwbio, Beijing, China) according to the manufacturer’s instruction. Real-time RT-PCR amplification was performed using an Ultra SYBR mixture kit (Cwbio) on the C1000 Touch Thermal Cycler (Bio-Rad, Hercules, CA, USA), and the results were analyzed using CFX Manager software (Bio-Rad). Target gene expression levels were quantified using the formula 2^−ΔΔct^ method [30]. Primer sequences (Invitrogen Corporation, Shanghai, China) and optimal PCR annealing temperatures are listed in Table 3.

### 4.5. Western Blot Analysis

Cells were lysed with RIPA (1% Triton X-100, 150 mM NaCl, 25 mM Tris pH 7.4, 5 mM EDTA, 0.5% sodium deoxycholate, 0.1% SDS, 5 mM tetrasodium pyrophosphate, 50 mM sodium fluoride, 1 mM Na_3_VO_4_, 2 mM phenylmethanesulfonyl fluoride, 0.076 U/mL aprotinin). After centrifugation at 13,800× *g* for 15 min, the supernatants were collected. Protein content was determined using BCA Protein Assay. Equal amounts of protein were subjected to SDS-PAGE. Proteins were electrophoretically transferred to PVDF membranes (Bio-Rad), and blocked with phosphate buffered saline/0.05% Tween-20 (PBS-T) containing 5% non-fat dry milk or BSA at room temperature (RT) for 1 h. Membranes were incubated with primary antibody TLR2, MyD88 (Abcam, Cambridge, MA, USA), p-p38 MAPK, p38 MAPK, p-NF-κB or NF-κB (Cell Signaling Technology, Boston, MA, USA) at a 1:1000 dilution in PBS-T at 4 °C overnight, washed four times with PBS-T, and then incubated with horseradish peroxidase (HRP)-conjugated secondary antibody (Beyotime Institute of Biotechnology) in PBS-T at room temperature for 1 h. Protein was detected using an enhanced chemoluminiscence (ECL) kit (Tiangen, Beijing, China).

### 4.6. RNA Interference (RNAi) of Gene TLR2

For gene silencing assays, the small interfering RNA of TLR2 (siTLR2) gene and negative control siRNA (siNC) were synthesized by Invitrogen Corporation. The sense and anti-sense strands of siRNAs were: siRNA 1, 5′-GGUGAAACAAAUUCAUUGATT-3′ (sense), 5′-UCAAUGAAUUUGUUUCACCTT-3′ (antisense); siRNA 2, 5′-CCUCUCUACAAACUUUAAUTT-3′ (sense), 5′-AUUAAAGUUUGUAGAGAGGTT-3′ (antisense); siRNA 3, 5′-GCAACUCAAAGAACUUUAUTT-3′ (sense), 5′-AUAAAGUUCUUUGAGUUGCTT-3′ (antisense); hepatocytes were transiently transfected with oligofectamine according to the manufacturer’s protocol in the presence or absence of 60 nM siRNA duplex. Western blot analysis of protein expression level of TLR2 was performed to identify inhibitory effect of siRNA in HH4 cell. After 48 h transfection with indicated siRNA, cells were treated with 5 mM FAC for 24 h and then the expression of a series of inflammation-related genes was detected using quantitative real-time RT-PCR or Western blot assay.

### 4.7. Statistical Analysis

Results were analyzed by one-way analysis of variance (ANOVA) or 2-sample unpaired *t*-test using Prism 5 program (Version 5.0). Differences in results were considered significant when *p* values were ≤ 0.05. Results are given as mean ± SEM of at least 3 independent experiments.

## 5. Conclusions

This is the first global proteomic analysis that compares the protein signatures of FAC-exposed HH4 cells with normal HH4 cells. Our study results suggest differentially expressed proteins are involved in multiple biological process including endocytosis, di-, tri-valent inorganic cation homeostasis, response to wounding, inflammatory response, anti-apoptosis, apoptotic mitochondrial changes and other processes. In addition, this work suggests for the first time that FAC overload can induce TLR2-mediated acute inflammatory response and activate IL6 expression via NF-κB pathways in HH4 cells. Our results should provide a theoretical basis for drug therapy of iron-associated liver damage.

## Figures and Tables

**Table 1:** Proteins differentially expressed in response to iron overload in HH4 cells.

Ratio ^1^	Protein IDs	Protein Description	Gene Name	MW [kDa] ^2^
−1.781	Q92896	Golgi apparatus protein 1	GLG1	135
−1.602	Q9NZN4	EH domain-containing protein 2	EHD2	61
−1.437	P09972	Fructose-bisphosphate aldolase C	ALDOC	39
−1.091	P42126	Isoform 2 of Enoyl-CoA delta isomerase 1, mitochondrial	ECI1	31
−1.09	P07996	Thrombospondin-1	THBS1	129
−0.983	P09603	Macrophage colony-stimulating factor 1	CSF1	60
−0.977	Q92522	Histone H1x	H1FX	22
−0.956	O95197	Isoform 3 of Reticulon-3	RTN3	26
−0.927	P21399	Cytoplasmic aconitate hydratase	ACO1	98
−0.895	Q15418	Isoform 3 of Ribosomal protein S6 kinase α-1	RPS6KA1	73
−0.845	P07203	Glutathione peroxidase 1	GPX1	22
−0.814	Q8TED1	Probable glutathione peroxidase 8	GPX8	24
−0.793	Q16678	Cytochrome P450 1B1	CYP1B1	61
−0.768	Q6EMK4	Vasorin	VASN	72
−0.749	P51580	Thiopurine S-methyltransferase	TPMT	28
−0.745	Q969V3	Isoform 2 of Nicalin	NCLN	63
−0.733	O00622	Protein CYR61	CYR61	42
−0.729	P06865	β-Hexosaminidase	HEXA	58
−0.7	P04179	Superoxide dismutase [Mn], mitochondrial	SOD2	25
−0.693	P17342	Isoform 2 of atrial natriuretic peptide receptor 3	NPR3	60
−0.685	P07602	Proactivator polypeptide	PSAP	58
−0.661	O43865	Putative adenosylhomocysteinase 2	AHCYL1	59
−0.657	P52630	Signal transducer and activator of transcription 2	STAT2	97
−0.657	P51553	Isocitrate dehydrogenase [NAD] subunit gamma, mitochondrial	IDH3G	43
−0.648	P61970	Nuclear transport factor 2	NUTF2	14
−0.647	Q9ULZ3	Apoptosis-associated speck-like protein containing a CARD (PYD and CARD domain containing)	PYCARD	22
−0.639	P30626	Sorcin	SRI	18
−0.637	P0DMM9	Sulfotransferase 1A3	SULT1A3	34
−0.617	Q9H845	Acyl-CoA dehydrogenase family member 9, mitochondrial	ACAD9	69
−0.615	P53634	Dipeptidyl peptidase 1	CTSC	52
−0.613	Q6P587	Acylpyruvase FAHD1, mitochondrial	FAHD1	25
−0.609	P16403	Histone H1.2	HIST1H1C	21
−0.602	P62805	Histone H4	HIST1H4A	11
−0.59	P04844	Isoform 2 of Dolichyl-diphosphooligosaccharide—protein glycosyltransferase subunit 2	RPN2	68
−0.588	Q13228	Selenium-binding protein 1	SELENBP1	52
0.586	P05362	Intercellular adhesion molecule 1	ICAM1	58
0.597	Q9Y6H1	Coiled-coil-helix-coiled-coil-helix domain-containing protein 2, mitochondrial	CHCHD2	16
0.601	Q9Y371	Endophilin-B1	SH3GLB1	41
0.61	P05161	Ubiquitin-like protein ISG15	ISG15	18
0.614	P31689	DnaJ homolog subfamily A member 1	DNAJA1	45
0.614	P11387	DNA topoisomerase 1	TOP1	91
0.621	P31153	S-adenosylmethionine synthase isoform type-2	MAT2A	44
0.636	Q14498	Isoform 3 of RNA-binding protein 39	RBM39	57
0.636	O75534	Isoform Short of Cold shock domain-containing protein E1	CSDE1	86
0.643	P02786	Transferrin receptor protein 1	TfR1	85
0.653	O75976	Carboxypeptidase D	CPD	153
0.656	Q9BQ52	Zinc phosphodiesterase ELAC protein 2	ELAC2	92
0.67	Q9UI12	ATPase, H^+^ transporting, lysosomal 50/57kDa, V1 subunit H, isoform CRA_c	ATP6V1H	52
0.678	P48729	Casein kinase 1, alpha 1, isoform CRA_g	CSNK1A1	38
0.681	P08473	Neprilysin	MME	86
0.685	Q9H2H9	Sodium-coupled neutral amino acid transporter 1	SLC38A1	54
0.692	P08195	Isoform 2 of 4F2 cell-surface antigen heavy chain	SLC3A2	58
0.702	Q9H5Q4	Dimethyladenosine transferase 2, mitochondrial	TFB2M	45
0.712	P29317	Ephrin type-A receptor 2	EPHA2	108
0.714	O14786	Neuropilin-1	NRP1	101
0.723	P11279	Lysosome-associated membrane glycoprotein 1	LAMP1	39
0.726	Q96PD2	Discoidin, CUB and LCCL domain-containing protein 2	DCBLD2	85
0.747	P16070	CD44 antigen (Fragment)	CD44	20
0.754	P46734	Dual specificity mitogen-activated protein kinase kinase 3	MAP2K3	39
0.765	P08754	Guanine nucleotide-binding protein G(k) subunit alpha	GNAI3	41
0.766	Q9H5V8	CUB domain-containing protein 1	CDCP1	93
0.777	Q13641	Trophoblast glycoprotein	TPBG	46
0.809	P02795	Metallothionein-2	MT2A	6
0.811	Q14978	Nucleolar and coiled-body phosphoprotein 1	NOLC1	74
0.825	P52926	High mobility group protein HMGI-C	HMGA2	11
0.833	P08962	CD63 antigen	CD63	16
0.837	P32455	Interferon-induced guanylate-binding protein 1	GBP1	68
0.837	P04183	Thymidine kinase, cytosolic	TK1	25
0.848	Q16831	Uridine phosphorylase 1	UPP1	34
0.858	P21980	Protein-glutamine gamma-glutamyltransferase 2	TGM2	69
0.875	P35080	Profilin-2	PFN2	10
0.882	P13726	Tissue factor	F3	33
0.885	P56945	Breast cancer anti-estrogen resistance protein 1	BCAR1	93
0.891	O15118	Niemann-Pick C1 protein	NPC1	142
0.892	Q14669	E3 ubiquitin-protein ligase TRIP12	TRIP12	220
0.92	Q12996	Cleavage stimulation factor subunit 3	CSTF3	83
0.935	Q9H4A6	Golgi phosphoprotein 3	GOLPH3	34
0.942	Q16850	Isoform 2 of lanosterol 14-α demethylase	CYP51A1	46
0.962	P11802	Cyclin-dependent kinase 4	CDK4	34
0.97	Q9Y3E0	Vesicle transport protein GOT1B	GOLT1B	14
0.975	Q9Y3D8	Transcription initiation factor TFIID subunit 9	AK6	17
0.983	Q9UBB6	Isoform 2 of neurochondrin	NCDN	77
1.003	P17844	Probable ATP-dependent RNA helicase DDX5	DDX5	107
1.028	P05120	Plasminogen activator inhibitor 2	SERPINB2	47
1.085	P11234	Ras-related protein Ral-B	RALB	23
1.127	Q96QD8	Sodium-coupled neutral amino acid transporter 2	SLC38A2	56
1.162	P52292	Importin subunit alpha-1	KPNA2	58
1.539	P40189	Interleukin-6 receptor subunit beta	IL6ST	104
1.54	Q06481	Amyloid-like protein 2	APLP2	76
1.742	P05067	Gamma-secretase C-terminal fragment 59	APP	81
2.309	P09914	Interferon-induced protein with tetratricopeptide repeats 1	IFIT1	55
2.408	P02787	Serotransferrin	TF	78
2.809	O60603	Toll-like receptor 2	TLR2	90

^1^: LOG (FAC/Veh, 2); ^2^: MW: relative molecular mass (MW) generated by the MS system.

**Table 2:** Differentially expressed proteins involved in various iron overload-induced biological process in HH4 cells.

Biological Process	Related Gene
Response to wounding	CD44; F3; DCBLD2; GPX1; MAP2K3; NRP1; SERPINB2; SOD2; THBS1; TLR2; TF; TfR1
Di-, tri-valent inorganic cation homeostasis	ACO1; APP; APLP2; IL6ST; MT2A; SRI; SOD2; TF; TfR1; TGM2
Regulation of growth	CD44; AK6; APP; CSF1; CYR61; DCBLD2; HMGA2; IDH3G; NRP1; BCAR1
Positive regulation of signal transduction	F3; CSF1; GPX1; GOLPH3; GOLT1B; IL6ST; THBS1; TGM2
Anti-apoptosis	SH3GLB1; F3; GPX1; SERPINB2; SOD2; THBS1; TGM2
Inflammatory response	CD44; F3; MAP2K3; THBS1; TLR2; TF; TfR1
Positive regulation of cell migration	F3; CSF1; ICAM1; IL6ST; BCAR1; THBS1
Endocytosis	ATP6V1H; EHD2; NPC1; APP; THBS1; TfR1
Iron ion homeostasis	ACO1; SRI; SOD2; TF; TfR1
Response to hypoxia	ALDOC; SOD2; THBS1; TF; TfR1
Positive regulation of cytokine production	PYCARD; IL6ST; THBS1; TLR2
Apoptotic mitochondrial changes	SH3GLB1; GPX1; SOD2
Positive regulation of NF-κB transcription factor activity	PYCARD; ICAM1; TLR2

**Table 3:** Forward and reverse primer sequences, annealing temperature and product size for different genes.

Gene	Primer	Sequence (5’–3’)	Product Size (bp)	Annealing Temperature (°C)
IL6ST	Forward	GTGAGTGGGATGGTGGAAGG	78	60
	Reverse	CAAACTTGTGTGTTGCCCATTC		
APP	Forward	GCCCTGCGGAATTGACAAG	144	60
	Reverse	CCATCTGCATAGTCTGTGTCTG		
TF	Forward	GGTGGCAGAGTTCTATGGGTC	172	60
	Reverse	ACAGTAAAGTAAGCCTATGGGGA		
TLR2	Forward	ATCCTCCAATCAGGCTTCTCT	118	60
	Reverse	GGACAGGTCAAGGCTTTTTACA		
MME	Forward	GATCGCACTCTATGCAACCTAC	83	60
	Reverse	TGTTTTGGATCAGTCGAGCAG		
TfR1	Forward	ACCATTGTCATATACCCGGTTCA	219	60
	Reverse	CAATAGCCCAAGTAGCCAATCAT		
CHCHD2	Forward	ACACATTGGGTCACGCCATTA	201	60
	Reverse	GCACCTCATTGAAACCCTCACA		
GLG1	Forward	CCAAGATGACGGCCATCATTT	103	60
	Reverse	AGCCGAATACTGCCACATTTC		
THBS1	Forward	TGCTATCACAACGGAGTTCAGT	108	60
	Reverse	GCAGGACACCTTTTTGCAGATG		
EHD2	Forward	TCCGCAAACTCAACCCTTTC	78	60
	Reverse	TCTCCAGGACCTGATTAGGGA		
CYP1B1	Forward	ACGTACCGGCCACTATCACT	142	60
	Reverse	CTCCCCACGACCTGATCCA		
TfR2	Forward	AATGTCCTGAGTCAGAGCGG	141	55.9
	Reverse	GGCAGACTCTACTGGAGCGA		
IL6	Forward	CCAGAGCTGTGCAGATGAGT	99	57.8
	Reverse	CATTTGTGGTTGGGTCAGG		
STAT3	Forward	ACACACGGTACCTGGAGCAG	172	57.8
	Reverse	TACTGCTGGTCAATCTCTCCC		
Ferritin	Forward	ATCAACCTGGAGCTCTACGC	155	57.8
	Reverse	TGGTTCTGCAGCTTCATCAG		
HAMP	Forward	CTGTTTTCCCACAACAGAC	230	59.8
	Reverse	CCTTCCTTATTTATTCCTGC		
β-actin	Forward	CTTCCTGGGCATGGAGTC	232	60
	Reverse	GCCGATCCACACGGAGTA		
